# Supplementary figures and images for: Basic Properties of Adipose-Derived Mesenchymal Stem Cells of Rheumatoid Arthritis and Osteoarthritis Patients
Source: Pharmaceutics. 2023 Mar 20;15(3):1003. doi: 10.3390/pharmaceutics15031003 (PMC10051260; doi:10.3390/pharmaceutics15031003)

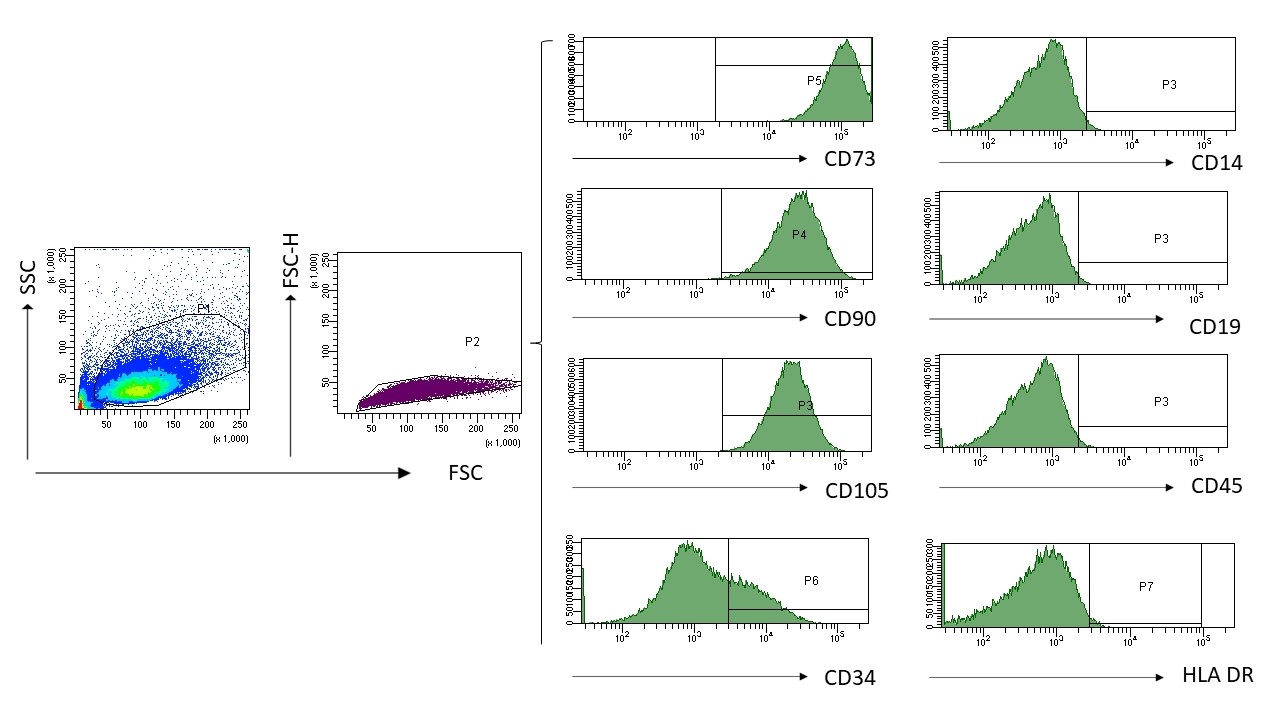

Supplement: Supplementary file 1 [file pharmaceutics-15-01003-s001.zip › supp figures/sup Figure S1.jpg]

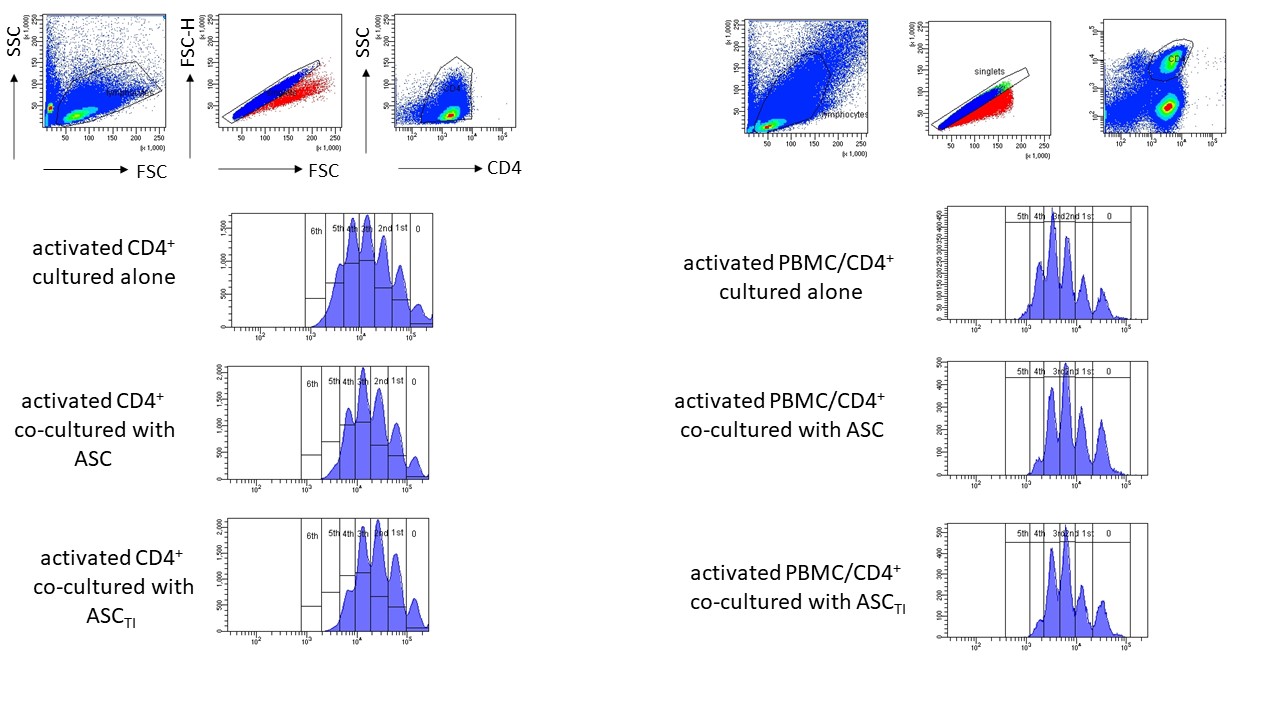

Supplement: Supplementary file 1 [file pharmaceutics-15-01003-s001.zip › supp figures/sup Figure S2.jpg]
